# An Important Movement

**Published:** 1899-08

**Authors:** 


### An Important Movement.

At a recent meeting of the National Association of Dental Faculties a resolution was offered and unanimously passed creating a Commission, whose duty it should be to consult and advise with all promoters of new colleges. And when such Commission should find that a projected college is not needed in its proposed locality, nor established with proper motives, nor with sufficient resources, nor to be conducted by men well equipped for the work, it shall be their duty to discourage such organizations.

They would simply advise against putting into operation such colleges, and they could at least suggest that colleges in any of these respects could hardly hope for membership in the national body. This Commission, if it proceeds wisely, can be the means of preventing the existence of colleges not needed and encouraging such as may be really needed. This Commission consists of Drs. Truman W. Brophy, Edward C. Kirk, and Albert H. Fuller.

